# Molecular Mechanics of the Moisture Effect on Epoxy/Carbon Nanotube Nanocomposites

**DOI:** 10.3390/nano7100324

**Published:** 2017-10-13

**Authors:** Lik-ho Tam, Chao Wu

**Affiliations:** School of Transportation Science and Engineering, Beihang University, 37 Xueyuan Road, Beijing 100191, China; leo_tam@buaa.edu.cn

**Keywords:** carbon nanotube, composite, moisture, mechanical property, molecular dynamics simulation

## Abstract

The strong structural integrity of polymer nanocomposite is influenced in the moist environment; but the fundamental mechanism is unclear, including the basis for the interactions between the absorbed water molecules and the structure, which prevents us from predicting the durability of its applications across multiple scales. In this research, a molecular dynamics model of the epoxy/single-walled carbon nanotube (SWCNT) nanocomposite is constructed to explore the mechanism of the moisture effect, and an analysis of the molecular interactions is provided by focusing on the hydrogen bond (H-bond) network inside the nanocomposite structure. The simulations show that at low moisture concentration, the water molecules affect the molecular interactions by favorably forming the water-nanocomposite H-bonds and the small cluster, while at high concentration the water molecules predominantly form the water-water H-bonds and the large cluster. The water molecules in the epoxy matrix and the epoxy-SWCNT interface disrupt the molecular interactions and deteriorate the mechanical properties. Through identifying the link between the water molecules and the nanocomposite structure and properties, it is shown that the free volume in the nanocomposite is crucial for its structural integrity, which facilitates the moisture accumulation and the distinct material deteriorations. This study provides insights into the moisture-affected structure and properties of the nanocomposite from the nanoscale perspective, which contributes to the understanding of the nanocomposite long-term performance under the moisture effect.

## 1. Introduction

Polymer nanocomposites comprise the reinforcements distributed in a matrix, which have attracted substantial interest in both academic and industrial communities. The nanocomposites reinforced by the nanoparticles in the form of the sphere, cylinder, and plate possess enhanced material properties, such as light weight, high stiffness, increased fatigue resistance, good chemical stability, and improved heat and thermal resistance [1]. In recent years, these nanocomposites have been increasingly used in a wide array of engineering fields across multiple length scales, ranging from the microscopic sensors and actuators in the micro-electromechanical systems, to the macroscopic composite materials in the aerospace, automotive, and construction industries [2,3,4]. In practice, a wide variety of the polymer matrix and the reinforcements have been used for fabricating the nanocomposite. Particularly, the epoxy is one of the most commonly used polymers, which possesses a three-dimensional cross-linked network formed by the cross-linking process occurred in the molecular level [5]. Meanwhile, due to the remarkable mechanical properties including the high elastic modulus and tensile strength, the carbon nanotube (CNT) is regarded as one of the most promising reinforcement materials [1]. The incorporation of a small amount of the CNT can substantially improve the mechanical properties of the resulting polymer nanocomposites, which can sustain the large degree of the elastic deformation, and ensure the durability of the engineering applications. However, as the epoxy is sensitive to the environment humidity, the moisture diffuses into the nanocomposite and interacts with the structure, which influences the mechanical performance of the nanocomposite [6,7]. For these reasons, the structural integrity of the epoxy nanocomposites is significantly affected in the moist environment. 

As the moisture-affected structural integrity of the polymer composites is a matter of concern during the intended service life, several experimental studies have been carried out to study the composite material under the influence of the moisture [8,9,10,11,12,13]. The diffusion and absorption of the moisture into the composite have been successfully obtained through the gravimetric measurements at different moisture conditioning durations [8,9,10,11,12]. With the incorporation of the reinforcement, it is reported that the saturated moisture concentration of the composite is either lower than, or close to, that of the neat polymer. The moisture absorption of the composite material is affected by the reinforcement, which also has an influential role in the moisture-affected material properties. Particularly, a recent experimental investigation of the epoxy/CNT composite has shown that the storage moduli decreases with increasing moisture ingression, and the value for the composite is higher when compared to the neat polymer after the same conditioning time [11]. Similar moisture-affected deterioration has been observed in the glass transition temperature of the epoxy/organoclay composite [8] and the shear stress of the epoxy/CNT composite adhesive obtained from the lap shear tests [13]. The lower degree of the degradation in case of the composite is attributed to the existence of the stiff reinforcement, which might affect the structure and the molecular interaction inside the composite under the wet condition. So far, the molecular origin of the moisture-affected property variations of the polymer composite is still unclear, such as the distribution, structure and molecular interactions of the water molecules inside the structure. 

**Figure 1:**
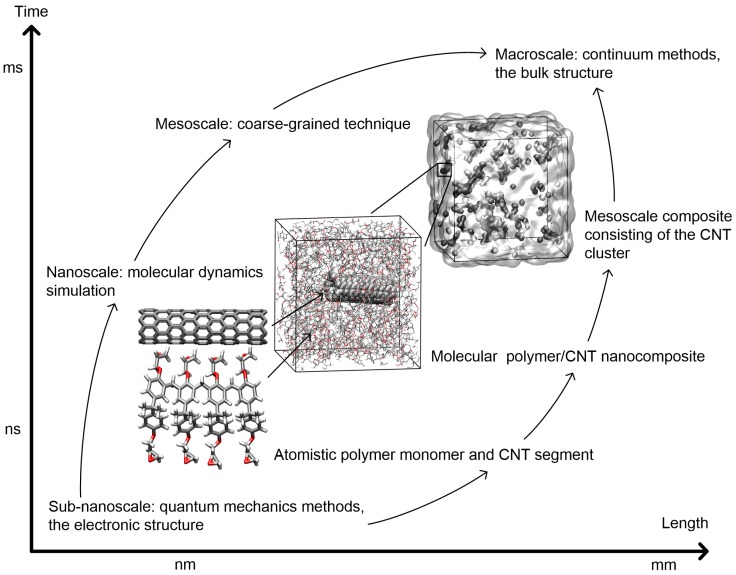
Overview of the polymer composite at different scales: from the atomistic building blocks of the polymer monomer and the carbon nanotube (CNT) segment, to the molecular model of the polymer/CNT nanocomposite, and to the mesoscale structure consisted of the CNT cluster. To understand the origin of the moisture effect, it is important to study the material behavior in the molecular level, so as to predict the system performance at the larger levels.

The polymer composite features the hierarchical structure ranging from the atomistic building blocks, including the polymer monomer and the CNT segment, to the molecular model with the CNT segment embedded in the polymer matrix, and to the mesoscale structure consisting of the CNT cluster, as shown in Figure 1. To understand the origin of the moisture effect, it is important to study the material behavior in the molecular level, so as to predict the system performance at the larger length and time scales. Molecular dynamics (MD) simulation has been considered as a fundamental and powerful technique for studying the molecular interaction inside the structure [14,15,16,17,18], and it has been widely used to investigate the structure and properties of the polymer, the carbon material, and the polymer composite at the nanoscale [19,20,21,22,23]. Microscopic information about the moisture diffusion and absorption inside the polymer and the nanocomposite has been obtained through observing the molecular motions [24,25,26]. The mechanism of the water molecule diffusion in the polymer is reported recently, where the water molecules absorbed in the structure can move between the microcavities and form hydrogen bonds (H-bonds) with the network polar groups [24]. Further simulation investigations have provided the information about the interactions among the polymer, the CNT, and the water molecules [27,28,29,30]. Notably, an atomistic simulation of the epoxy network has quantitatively shown that at the low moisture concentration, most of the water molecules tend to form H-bonds with the structure functional groups, while forming bonds with other water molecules at the high concentration, but the detailed H-bond network inside the structure is not provided [27]. Furthermore, the MD simulation has been adopted for investigating the moisture effect on the properties of the epoxy and epoxy-based composite [20,26]. Particularly, the adhesion energy of the epoxy/nanoclay composite to the substrate is significantly reduced when the water molecule is added at the interface close to the substrate, but the mechanism of the moisture effect is not clear [26]. These MD studies have advanced the understanding of the moisture effect on the polymer, but until now, very few investigations have been focused on the relationship between the molecular interaction among the polymer nanocomposite and the absorbed water molecules and the structure and properties of the nanocomposite. Such knowledge enables one to understand more in-depth about the molecular origin of the moisture-affected variation in the structure and properties of the epoxy/CNT nanocomposite.

The objectives of this study are to understand the molecular interactions between the water molecules and the epoxy/CNT nanocomposite in the molecular level, so as to evaluate the effect of the moisture on the structure and properties of the nanocomposite, and to predict the durability of the related engineering applications at the larger scales. The epoxy nanocomposite reinforced by a CNT segment is modeled by using a dynamic cross-linking algorithm, which is applicable to the construction of the epoxy-based materials involving the highly cross-linked network [19]. To mimic the different moisture absorption states in reality, the constructed model is solvated with a successive amount of the water molecules. Through examining the local structure and the H-bond network in the systems, the molecular interactions between the nanocomposite structure and the added water molecule are characterized, which are correlated with the mechanical behavior of the solvated structures. This paper provides the knowledge on the structure and properties of the epoxy/CNT nanocomposite with various moisture concentrations, which forms the fundamental basis for predicting the structural integrity of the nanocomposite during the intended service life, as well as for developing better products with improved moisture barrier properties.

## 2. Results and Discussion

The polymer matrix used in this study is the cross-linked epoxy, and its cross-linking process and physical properties have been investigated in our previous atomistic investigation [19]. The epoxy monomer consists of four components of the diglycidyl ether of bisphenol A, which are connected by the methylene group, as shown in Figure 1. For the CNT filler, it is reported that the chirality and diameter of the single-walled CNT (SWCNT) have limited effects on its Young’s modulus [31]. In this study, an armchair (5,5) SWCNT segment with a diameter of 6.8 Å is selected as a representative model of the reinforcement, and the SWCNTs with the similar diameter have been synthesized in the experiment [32,33]. According to various experimental and simulation studies, the mass fraction of the CNT in the composite is usually designed to be less than 10.0 wt% [10,11,12,13,34,35]. Accordingly, the SWCNT segment with a length of 24.6 Å is used in the simulation, as shown in Figure 1, and its mass fraction in the composite equals 4.3 wt%. Meanwhile, it should be noted that the SWCNT length is less than that of the periodic simulation cell edge of around 4.5 nm. Though the length of the CNT is several orders of magnitude shorter than the bulk material, the CNT reinforcing effect on the nanocomposite has been observed as equal to that in experiments, which indicates that the short aspect ratio SWCNT segment is reasonable as the filler in the nanocomposite modeling [35,36]. The MD simulation results based on the molecular model of the epoxy/SWCNT nanocomposite as shown in Figure 1 are presented here, including the validation of the nanocomposite model and the discussions about the moisture effect on the structure and properties of the epoxy/SWCNT nanocomposite from the nanoscale perspective.

### 2.1. Structure and Properties of the Epoxy/SWCNT Nanocomposite

The epoxy/SWCNT nanocomposite model with no moisture is validated first by determining its physical properties, i.e., the density (*ρ*) and the Young’s modulus (*E*), and comparing these against the experimental data. During the structural equilibration, the system is equilibrated under the atmospheric pressure for 5 ns and the three orthogonal directions are adjusted independently. The *ρ* of the modeled nanocomposite structure is sampled at a 2 ps interval, and the recorded data from the last 1 ns equilibration run is used for the calculation, such that the statistical error is minimized. With the addition of the SWCNT, the *ρ* of the nanocomposite model is measured to be 1.07 ± 0.002 g·cm^−3^, which is higher than the corresponding simulated value of the neat cross-linked epoxy in the range of 1.04 g·cm^−3^ to 1.05 g·cm^−3^ as reported in our previous work [19]. The increase of the composite density due to the addition of the CNT has been observed in various experimental and simulation studies, which can be attributed to the change in the cross-linked epoxy matrix, especially in the interfacial layer surrounding the CNT [37,38,39]. Specifically, previous simulation studies have demonstrated that a closely packed epoxy structure can be formed close to the epoxy-CNT interface, which leads to the increase of the nanocomposite density [38,39]. Another reason to the higher density is that the free volume of the epoxy matrix is decreased when the SWCNT is added. By using the Connolly surface algorithm, the free volume of the modeled structure is analyzed [40]. During the last 1 ns equilibration process, the fractional free volume of the model is sampled at a 200 ps interval. The measured fractional free volume of the epoxy and the nanocomposite is 23.85 ± 0.26% and 21.08 ± 0.41%, respectively. From these observations, it is found that the nanocomposite with the addition of the SWCNT possesses the structural characteristics close to the reported observation, i.e., the densified structure and the decrement in the structure free volume compared to the neat epoxy. 

After the structural characterization, the Young’s modulus of the nanocomposite model is quantified. The stress-strain data obtained from the tensile deformation of the nanocomposite model are shown in the Supplementary Materials (Figure S1), in comparison to those of the neat epoxy obtained from the experiment and simulation [41,42]. During the dynamic deformation, the stress of the nanocomposite model along the stretched direction shows a linear relation to the applied strain, which indicates that the structure is elongated elastically within this small-strain deformation. Based on the obtained stress-strain data, the *E* is measured by carrying out a linear regression analysis on the data, and the coefficient of determination for the fitted curve is over 0.99. The *E* of the nanocomposite model is measured to be 4.88 ± 0.07 GPa, revealing a significant increase in the stiffness in comparison to the neat cross-linked epoxy, with the experimental value ranging from 2.70 GPa to 4.02 GPa and the simulated results in the range of 2.67–4.43 GPa [19,41,42]. The enhancement of the nanocomposite Young’s modulus by the embedded CNT agrees closely with various measurements [1,7,22,23], which is due to the stiff CNT reinforcement and the efficient stress transfer from the epoxy matrix to the CNT. In view of the good agreement of the structural and mechanical properties of the modeled nanocomposite with the available data, the generated model of the epoxy/SWCNT nanocomposite is regarded as the reasonable structure close to those found in the real system, which is used as the basis in the following discussion.

### 2.2. Moisture Effect on the Structure and Properties of the Epoxy/SWCNT Nanocomposite

In order to study the moisture effect on the epoxy/SWCNT nanocomposite, the equilibrated structure is solvated with a successive amount of the water molecule, such that the different states of the moisture absorption are mimicked. The maximum moisture concentration of the epoxy nanocomposite is reported to be less than 4.0 wt% [10,11,12]. In this study, the moisture concentration of the nanocomposite model ranges from 1.0, 2.0, 3.0, to 4.0 wt% of the molar mass of the structure, and the concentration of 4.0 wt% is regarded as the saturated condition. Based on the modeled systems, the moisture effect is firstly characterized by examining the local structural changes in the nanocomposite with various moisture concentrations. The epoxy matrix of the nanocomposite includes two oxygen-containing functional groups, i.e., the hydroxyl groups and the ether oxygen atoms, which are important potential hydrogen bonding sites and tend to form the H-bonds among the functional groups and with the absorbed water molecules in the structure. Due to the polar interactions with the functional groups, the distribution of the water molecules in the nanocomposite structure can be influenced. In order to determine the water distribution, the arrangement of the functional groups around the SWCNT segment is firstly characterized. In general, the radial distribution function (RDF) is utilized to determine the normalized probability of finding an object at a distance *r* away from another object, which provides the useful information about the arrangement of the functional group. Here, the intermolecular RDFs between the oxygen of the functional groups and the carbon of the SWCNT are calculated and shown in Figure S2, with respect to the moisture concentration. The RDFs with similar shapes are reported for the polymer distribution in various nanocomposites [43,44]. For the RDFs of the hydroxyl groups, the small peaks at the distance of 2.5–5.0 Å indicate the favorable distribution of these functional groups in the interfacial layer surrounding the SWCNT. As the quantity of the hydroxyl groups in the structure is smaller than that of the ether groups, the sparse hydroxyl groups in the interfacial layer can aggregate at a certain distance due to the H-bond interaction and lead to the small peaks. With various moisture concentrations, the RDFs for the same functional group overlap, showing that the arrangement of the epoxy matrix are relatively the same for the different nanocomposite models, which is also observed from examining the orientation of the SU-8 monomers against the SWCNT axis.

**Figure 2:**
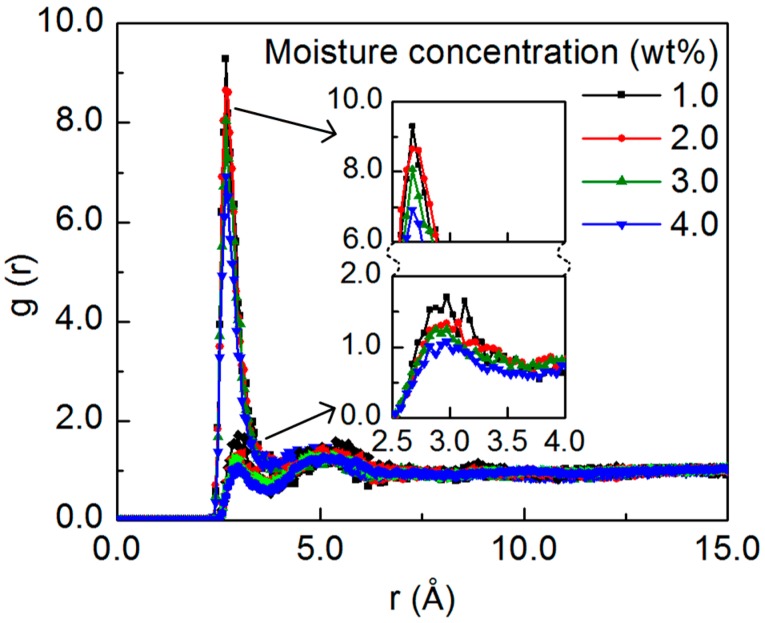
The radial distribution function (RDF) between the oxygen of the functional groups and the oxygen of the water molecule with respect to the moisture concentration. The RDFs for the water-hydroxyl interactions display higher peaks than those for the water-ether interactions, as demonstrated in the enlarged picture. Meanwhile, the intensity of the peak decreases monotonically with the increasing moisture concentration for both water-functional group interactions.

After characterizing the arrangement of the epoxy matrix around the SWCNT segment, the distribution of the added water molecules in relation to the functional groups is quantified. Specifically, the RDFs between the oxygen of the functional groups and the oxygen of the water molecule are calculated, as shown in Figure 2, with respect to the moisture concentration. All of the RDFs display a sharp peak at the distance between 2.5 Å to 3.5 Å, which features the presence of the H-bond interaction between the added water molecules and the functional groups. The higher peak of the water-hydroxyl RDFs indicates that the water molecules preferentially locate in the vicinity of the hydroxyl groups, as they are highly electrophilic in nature. Meanwhile, it can be seen that the intensity of the peak decreases monotonically with the increasing moisture concentration, which is due to the fact that the number of the functional groups in the system is constant, and the fraction of the added water molecules forming the H-bonds with the nanocomposite decreases with the increasing concentration, leading to the decrease in the RDF peak intensity. For comparison, the intermolecular RDFs for the neat epoxy structure are shown in the Supplementary Materials (Figure S3) [42]. The RDFs for the water-hydroxyl and water-ether interactions show similar shapes and positions as those for the nanocomposite structure, while the peak of the RDFs for the nanocomposite structure is relatively higher, indicating the water aggregation in the vicinity of the functional groups is more pronounced in the nanocomposite structure, which could be due to the existence of the hydrophobic SWCNT segment in the system, as discussed subsequently.

**Figure 3:**
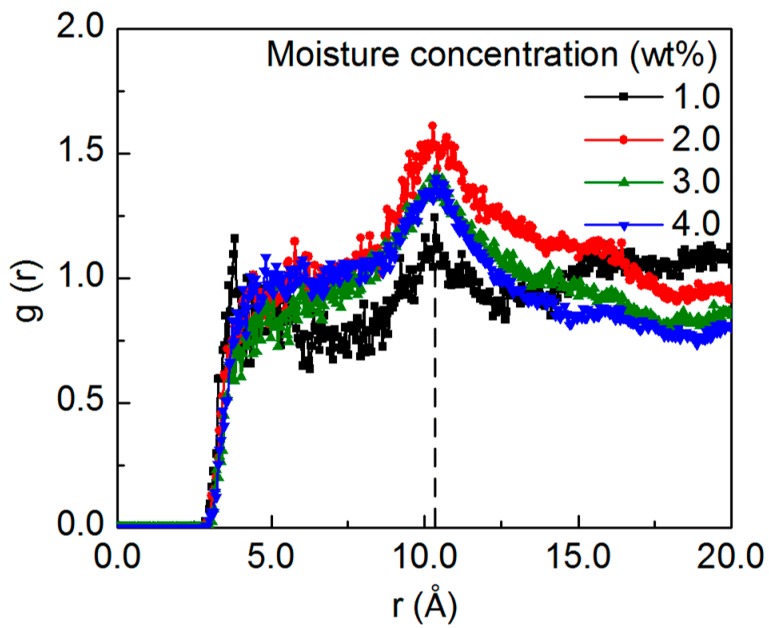
The RDF between the oxygen of the water molecules and the carbon of the SWCNT with respect to the moisture concentration. The peak at the distance of about 10.3 Å is resulted from the accumulation of the water molecules at the distance where the short-range van der Waals (vdW) interaction is cutoff, which is caused by the repulsive effect of the hydrophobic SWCNT.

Apart from the effect of the epoxy functional groups, the distribution of the water molecules can be affected by the SWCNT segment, which is characterized by measuring the water-SWCNT RDFs for the systems with different moisture concentrations, as shown in Figure 3. Meanwhile, the two-dimensional distribution map of the water molecules in the plane perpendicular to the SWCNT axis after the equilibration is shown in Figure S4, with respect to the moisture concentration. At the concentration of 1.0 wt%, the RDF shows a well-defined first peak around the distance of 3.8 Å and a second peak at the distance of about 10.3 Å. With the existence of the SWCNT segment, some water molecules can diffuse to the free volume in the epoxy-SWCNT interface, which leads to the first peak. Meanwhile, due to the repulsive effect of the hydrophobic SWCNT, the other water molecules are repulsed by the SWCNT through the short-range van der Waals (vdW) interaction and some of them are accumulated at the cutoff distance from the SWCNT surface, which leads to the second peak. With the increasing concentration, due to the limited available space in the interface, only a small portion of the added water molecules diffuse to the interface region, as demonstrated by the two-dimensional distribution map. Therefore, the peak close to the interface is not obvious at the high moisture concentrations. Specifically, at the 2.0 wt% concentration, the newly-added water molecules repulsed by the SWCNT tend to accumulate at the cutoff distance, leading to the noticeable peak intensity. When the moisture concentration continuously increases, as there is limited available space at the cutoff distance, the added water molecules tend to diffuse to other available space in the structure, and the peak intensity drops gradually. The simulation results suggest that the distribution of the added water molecules is affected by the functional groups and the embedded SWCNT in the structure, which varies with the moisture concentration. Furthermore, it is noted that there is a relatively high correlation among the distribution map of the water molecules with various concentrations, which is resulted from the successive addition of the water molecules. As the amount of 1.0 wt% water molecules is added successively to the equilibrated model for the condition with various concentrations, the newly-added water molecules are also affected by the water molecules existing in the structure, which can diffuse to the vicinity of the existing water molecules. Therefore, it leads to the relatively high correlation among the final distribution of the water molecules with various concentrations.

**Figure 4:**
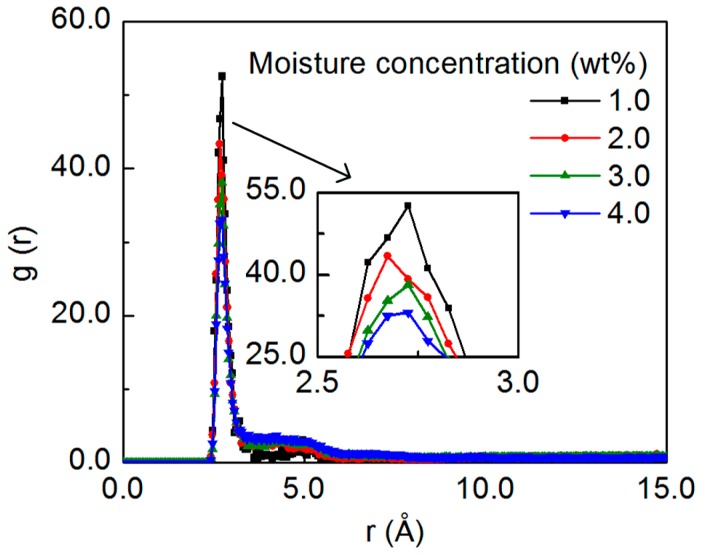
The RDF of the oxygen atoms in the water molecules with respect to the moisture concentration, with the highest peak height for the system with the concentration of 1.0 wt%.

With the knowledge of the preferential distribution of the water molecules in the nanocomposite, the structure of the water molecules in the system is characterized, by focusing on the water-water RDF for the systems containing various amounts of the water molecules, as shown in Figure 4. The water-water RDFs show the sharp peak at a distance of around 2.7 Å for all the systems, with the highest peak height for the system with the lowest concentration in this study, i.e., 1.0 wt%. The relationship between the water-water RDF peak and the moisture concentration is in good accord with previous MD simulation results, where the peak height of the water-water RDF decreases with the moisture concentration [45,46,47], and the RDF peak of the oxygen atoms in the bulk water is lower than that in the solvated polymer structures at any moisture concentration [47,48]. This observation demonstrates that the water molecules are more likely to be hydrogen bonded with each other and form the cluster at high moisture concentrations.

**Figure 5:**
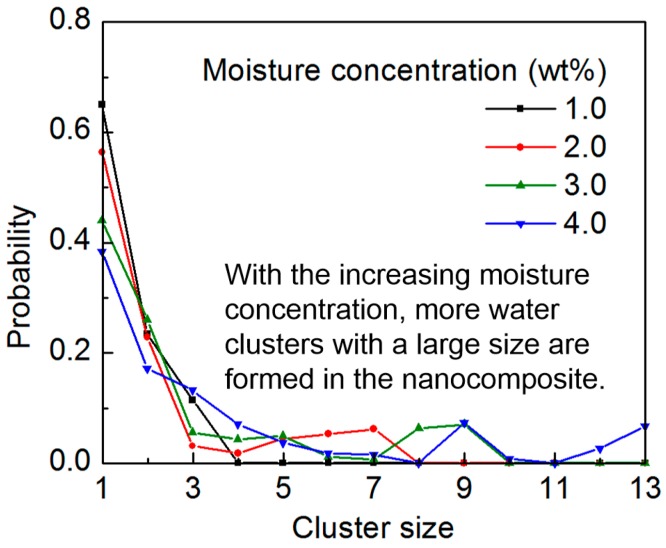
The cluster size distribution of the water molecules in the epoxy/SWCNT nanocomposite with respect to the moisture concentration: with the increasing moisture concentration, more water molecules are involved in forming the large clusters in the system.

**Figure 6:**
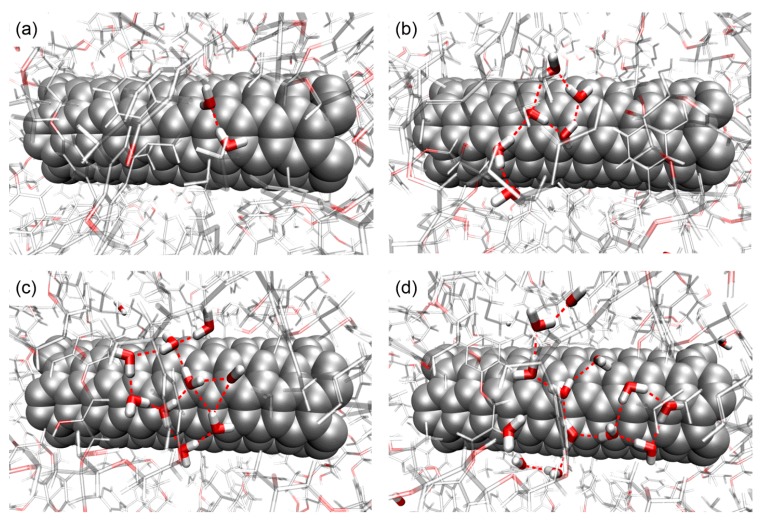
Simulation snapshots of the water cluster in the epoxy-SWCNT interface in the nanocomposite: the water molecules diffuse to the free volume in the epoxy-SWCNT interface, and form the clusters in the system: (**a**) 1.0 wt%; (**b**) 2.0 wt%; (**c**) 3.0 wt%; and (**d**) 4.0 wt%. For clarity, the epoxy structure is faded and the H-bond is indicated by the dash line.

Further information about the structure of the water molecules is obtained by quantifying the size probability of the water cluster. When the oxygen of the water molecules is less than a cutoff distance of 3.5 Å and is also within a cutoff angle of 50°, the water molecules are considered to be in the same cluster [49]. The probability of the cluster size is shown in Figure 5, with respect to the moisture concentration. It should be mentioned that the ordinate indicates the probability that a randomly-chosen water molecule will be in the cluster of a provided size. When the moisture concentration is low, a large portion of the water molecules exist as single molecules in the structure, with about 35% existing as pairs. When the moisture concentration increases, the water molecules tend to form clusters. The observation of the water clustering behaviors with different moisture concentrations is consistent with the recent observation in the polymer materials [28,50]. A close examination of the MD simulation process shows that some water molecules can diffuse to the free volume in the epoxy-SWCNT interface, as shown in Figure 6. With the increasing moisture concentration, more water molecules are involved in forming the large clusters in the system.

**Figure 7:**
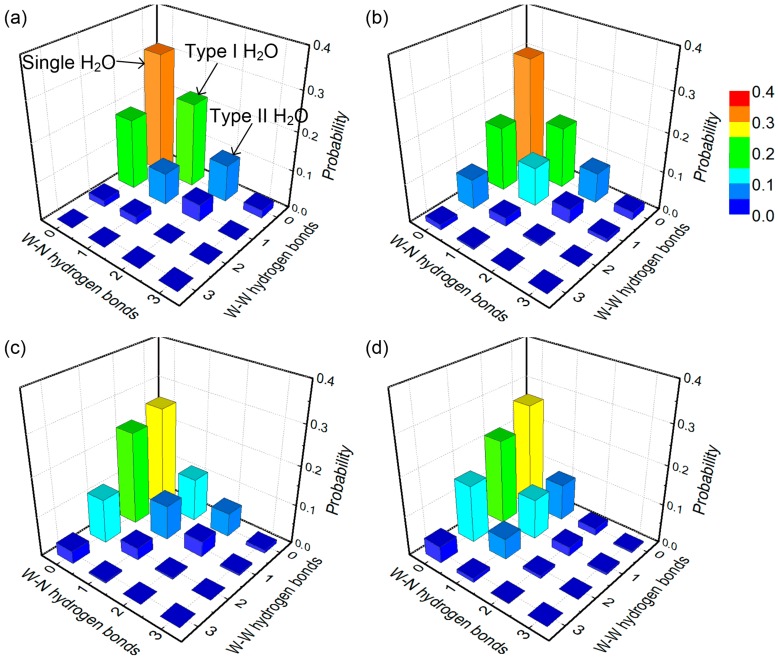
The distribution of the hydrogen bond (H-bond) probability for a given water molecule in the epoxy/SWCNT nanocomposite with respect to the moisture concentration: (**a**) 1.0 wt%; (**b**) 2.0 wt%; (**c**) 3.0 wt%; and (**d**) 4.0 wt%. The single water molecules do not form the H-bond, the Type I water molecules form one water-nanocomposite H-bond, and the Type II water molecules form more than one water-nanocomposite H-bond. The W-N and W-W denote the water-nanocomposite and the water-water, respectively.

Due to existence of the epoxy functional groups and the hydrophobic SWCNT, the preferential distribution and the structure of the water molecules in the nanocomposite vary with different moisture concentrations, which can affect the molecular interactions inside the structure. To gain insight into the interactions between the nanocomposite structure and the water molecules, the H-bond network is characterized, with the focus on the probability of forming the water-nanocomposite and water-water H-bond for a given water molecule, and the probability of forming the H-bond between the water cluster and the nanocomposite. The H-bond is defined based on two geometric criteria: the distance between the donor and the acceptor oxygen is less than 3.5 Å, and the donor-hydrogen-acceptor angle is above 130° [49]. The cutoff distance value corresponds to the position of the first minimum of the water-functional group RDFs as shown in Figure 2. Meanwhile, all of the potential hydrogen bonding sites in the nanocomposite structure are considered for determining the water-nanocomposite H-bond, including the oxygen and hydrogen of the hydroxyl groups and the oxygen of the ether groups. During the equilibration run, the H-bond network is monitored at a 100 ps interval, and the recorded data from the last 1 ns equilibration run is used for the calculation. The measured H-bond probability distribution for a given water molecule and for a given cluster is shown in Figure 7 and Figure S5, respectively, for different moisture concentration conditions.

**Figure 8:**
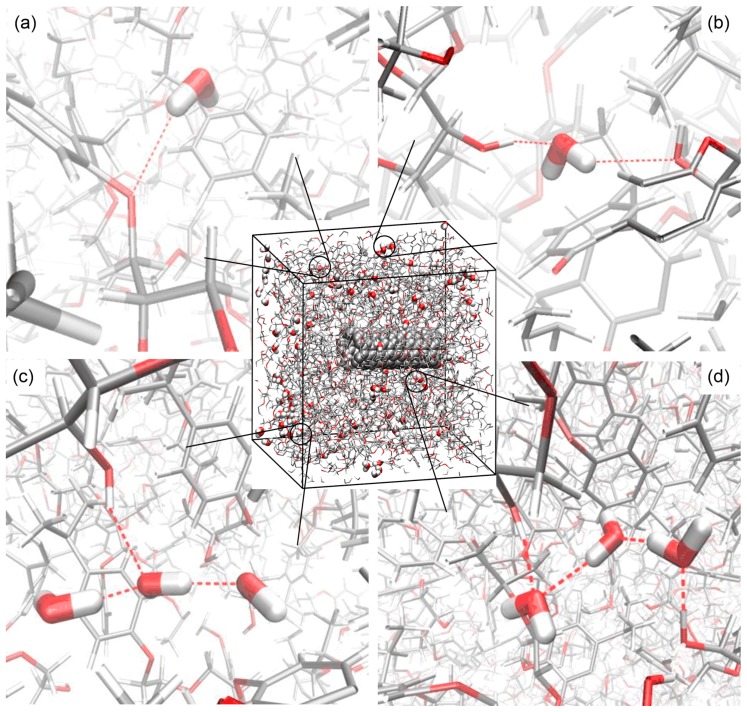
The schematic diagram of (**a**) the Type I water molecule forming one water-nanocomposite H-bond; (**b**) the Type II water molecule forming more than one water-nanocomposite H-bond; (**c**) the Type I water cluster; and (**d**) the Type II water cluster. For clarity, the epoxy structure is faded, the water cluster only consists of three water molecules, and the H-bond is indicated by the dashed line.

From these figures, it is observed that a large portion of the water molecules do not form H-bonds with the nanocomposite structure and other water molecules, existing as single water molecules. These single water molecules neither show strong interactions with the nanocomposite structure nor with other water molecules, and they can only weakly affect the mechanical properties of the nanocomposite with various moisture concentrations. Meanwhile, there are a certain number of water molecules forming one to three H-bonds with the nanocomposite, which have no H-bond with other water molecules. These water molecules can be classified into two types, as indicated in Figure 7 and Figure 8. The Type I water molecules forming one water-nanocomposite H-bond can interfere with the molecular interactions established by the vdW and Coulombic force inside the structure, leading to the material plasticization, as demonstrated in Figure 8a [6,51]. Comparatively, the Type II water molecules forming more than one H-bond with the nanocomposite can effectively form the bridge between the functional groups in the structure, leading to the secondary cross-linking process, as demonstrated in Figure 8b. When the moisture concentration increases, the added water molecules tend to form the H-bonds, especially with other existing water molecules. The water molecules interacting by the H-bonds form the water clusters, which can affect the molecular interactions inside the system and cause a deviation in the nanocomposite properties. Accordingly, these water clusters can be classified into two types, as indicated in Figure S5 and Figure 8. Among these water clusters, a portion of the water clusters form the H-bonds with the nanocomposite, being the Type I water cluster with one water-nanocomposite H-bond, or the Type II water cluster forming more than one water-nanocomposite H-bond, as demonstrated in Figure 8c,d, respectively. From the simulation results, it is observed that when there is a scarcity of water, the water molecules predominantly form H-bonds with the functional groups in the structure rather than with other water molecules. When the moisture concentration increases, the water molecules tend to form a larger number of the H-bonds with the existing water molecules rather than with the nanocomposite structure. 

The formation of the water cluster and its interactions with the nanocomposite structure vary with different moisture concentrations, which affect the movements of the water molecules. The diffusion of the water molecules is determined by examining the mean squared displacement (MSD) of the water molecules, as shown in Figure S6. It is observed that with the increasing concentration, the diffusion of the water molecules inside the structure is restricted, which is because more and more water molecules are involved in the H-bond interactions either with other water molecules or with the functional groups, and the dynamics of the water molecules reach the equilibrium state. The simulation results support the observation for the cluster formation and the H-bond analysis as discussed previously.

**Figure 9:**
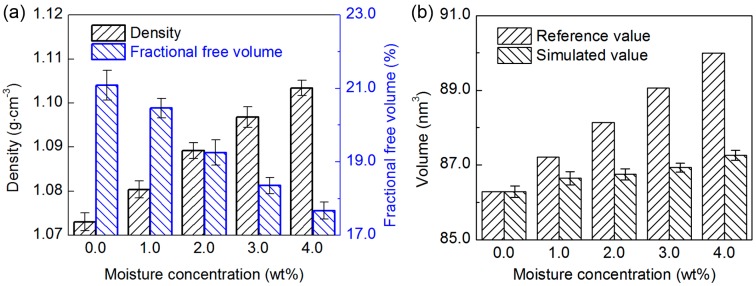
The (**a**) density and fractional free volume and (**b**) volume of the epoxy/SWCNT nanocomposite with respect to the moisture concentration at the temperature of 300 K and the pressure of 1 atm.

The structural variation under the moisture effect plays an influential role in the material properties of the epoxy/SWCNT nanocomposite. During the equilibration process, the *ρ* and volume of the nanocomposite model is recorded at a 2 ps interval, and the recorded data from the last 1 ns equilibration run is averaged. The measured *ρ* of the nanocomposite model with various moisture concentrations is shown in Figure 9a, with the error bar showing the standard derivation of the averaged recorded data. For the solvated nanocomposite systems, the *ρ* shows a monotonic increase with the moisture concentration, and reaches the maximum of 1.10 ± 0.002 g·cm^−3^ at the moisture concentration of 4.0 wt%. In order to understand the reason for such behavior, the measured volume of the nanocomposite is shown in Figure 9b. For comparison, the volume of the dry model and the corresponding water molecules at various concentrations are added together and regarded as the reference value. With the increasing moisture concentration, the volume increment of the nanocomposite model is smaller than that of the added water molecules, which indicates that the added water molecules mainly diffuse to the free volume and the vicinity of the existing water molecules in the structure, leading to the decrease of the fractional free volume, as shown in Figure 9a. With the limited expansion of the system and the weight gain by the added water molecules, it results in the monotonic increase of the nanocomposite density with the increasing moisture concentration. Furthermore, the simulation results show the moisture induced swelling of the nanocomposite, which can affect the structural integrity of the nanocomposite.

**Figure 10:**
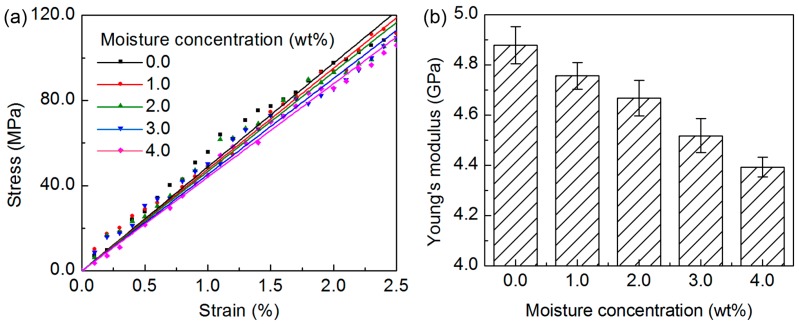
The (**a**) stress-strain data, and the (**b**) Young’s modulus of the epoxy/SWCNT nanocomposite obtained from the small-strain uniaxial tensile deformation at the temperature of 300 K and the pressure of 1 atm.

After determining the moisture effect on the volumetric properties, the moisture-affected elastic properties are now quantified by using the Young’s modulus as an indicator. The stress-strain data of the nanocomposite model with various moisture concentrations from the tensile deformations are averaged and shown in Figure 10, together with the calculated *E* and the error bar obtained from the linear regression of the averaged simulated data. For the solvated structures, the *E* decreases gradually to 4.39 ± 0.04 GPa for the structure with the maximum moisture concentration of 4.0 wt%, revealing a 9.9% reduction compared to the dry sample. Such moisture-affected deterioration in the mechanical properties is consistent with the experimental observations [8,11,13], which can be related to the variation in the structure and the molecular interactions in the nanocomposite due to the moisture absorption. When the moisture concentration is low, i.e., 1.0 wt% in this study, the water molecules are located in the vicinity of the functional groups and expand the nanocomposite structure. The water molecules and the small clusters affect the molecular interaction inside the structure by forming a significant number of water-nanocomposite H-bonds. The existence of the Type I water molecules and clusters plays a vital role in the plasticization, while the bridging of the Type II water molecules and clusters leads to a secondary cross-linking process, and the stiffening of the nanocomposite to a certain extent. Under the influence of these various factors, the *E* of the nanocomposite shows a marginally decrease of 2.5% at this low moisture concentration. When the concentration continuously increases, the water molecules preferably form a large number of the water-water H-bonds and the large water clusters. The water clusters are located in the vicinity of the epoxy functional groups and the available space in the epoxy-SWCNT interface, which can significantly disrupt the molecular interaction, and lead to the continuous decrease of the elastic modulus. The inferred trend of the mechanical properties with various moisture concentrations reported here is valid for the CNTs with a larger length at the nanometer scale.

**Figure 11:**
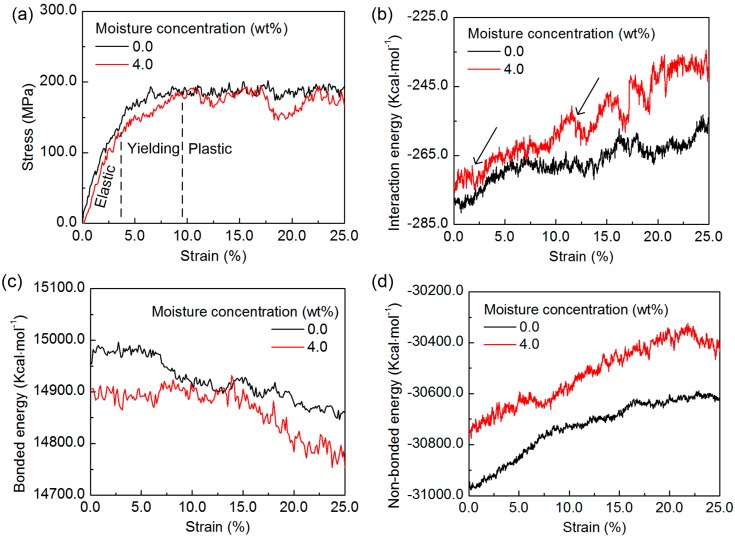
The (**a**) stress-strain curve and (**b**–**d**) the energy evolution of the epoxy/SWCNT nanocomposite during the tensile deformation to the plastic regime: (**b**) the interaction energy between the epoxy and the SWCNT (the arrows show the fluctuation during the elastic and plastic deformation regime); (**c**) the bonded energy, and (**d**) non-bonded energy of the nanocomposite.

**Figure 12:**
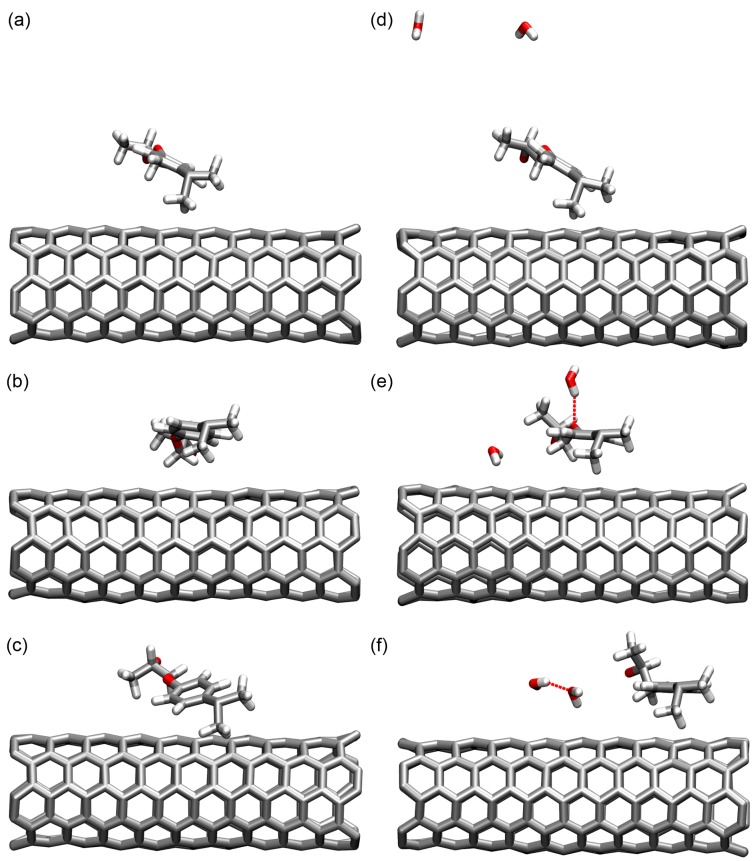
The configuration of the SWCNT segment and the selected epoxy chain in the nanocomposite with no moisture concentration when the strain is (**a**) 0.0%, (**b**) 13.0%, and (**c**) 25.0%; and with 4 wt% moisture concentration when the strain is (**d**) 0.0%, (**e**) 13.0%, and (**f**) 25.0%. The H-bond is indicated by the dashed line.

To further quantify the moisture-affected mechanical properties, the nanocomposite models with moisture concentrations of 0 and 4 wt% are subjected to the computational tensile deformation to the plastic regime. The stress-strain curves for the models are shown in Figure 11. After the initial elastic deformation stage, the structure yields and undergoes the plastic deformation, as similar to the stress-strain responses observed for different polymer/CNT composites [52,53,54]. During the plastic regime, the stress of the nanocomposite with no moisture fluctuates around a relatively constant level, which indicates that the nanocomposite structure is strained adequately. However, for the nanocomposite with the 4 wt% moisture concentration, the stress drops occasionally, implying that the existence of the water molecules interferes with the molecular interactions inside the structure and, thus, weakens the material performance. From the stress-strain curves, it is inferred that the ultimate strength and the toughness of the nanocomposite decreases with the addition of the water molecules, which is in accordance with the trend of the Young’s modulus. To understand the mechanism of the effect of the water molecules on the nanocomposite plastic deformation, the configuration of the structure is examined and shown in Figure 12, by selecting the SWCNT segment and the same epoxy chain for demonstration. It is observed that, with no water molecules in the structure, the epoxy chain can stick more firmly with the SWCNT segment during the tensile deformation, thus ensuring the efficient stress transfer from the epoxy matrix to the CNT, as shown in Figure 12a–c. With the addition of the water molecules, they can diffuse to the interface region and interact with the epoxy chain, and the strained epoxy matrix slides along the SWCNT surface more easily, as shown in Figure 12d–f. The slippage event occurs continuously and leads to the drop in stress. This observation can be further confirmed by examining the energy evolution of the nanocomposite structure during the deformation, as shown in Figure 11b–d. The value of the energy in the nanocomposite model is slightly different with the addition of the water molecules. With no water molecules, the bonded energy and non-bonded energy of the nanocomposite, as well as the interaction energy between the epoxy and the SWCNT change steadily, which indicates that the stress dissipation is mainly through the deformation within the epoxy matrix. With the addition of the 4 wt% moisture concentration, the evolution of the interaction energy between the epoxy and the SWCNT experiences relatively large fluctuations, during both the elastic and plastic deformation regime, as demonstrated in Figure 11b. This phenomenon further demonstrates that with the existence of the water molecules, the epoxy chain can slide along the SWCNT more easily, and it affects the non-bonded interaction between the epoxy matrix and the SWCNT, and the local high stress dissipation. The finding is in good accordance with recent observations, where the non-bonded interaction between the constituents in the nanocomposite plays an important role in dissipating the local high stress in the plastic deformation regime [55,56]. In addition, the bonded energy of the nanocomposite with 4 wt% moisture concentration evolves steadily at the beginning, which can be due to the reason that as the energy dissipation is mainly through the slippage between the epoxy matrix and the CNT, the strained epoxy matrix after the slippage reaches the local energy minimum state, thus maintaining the bonded energy.

From these simulation results, it is observed that, due to the existence of the functional groups and the hydrophobic SWCNT, the distribution and structure of the water molecules, as well as the interactions between the water molecules and the nanocomposite structure, are altered with various moisture concentrations, which significantly affect the structural integrity of the epoxy/SWCNT nanocomposite, such as the swelling and plasticization to a certain degree. The material degradation under the moisture effect agrees very well with the recent experimental studies of various epoxy-based nanocomposites [8,11,13]. When focusing at the nanoscale, the molecular model can mimic the mechanism of the moisture effect by capturing the variation in the interactions between the added water molecules and the nanocomposite structure, and the formation of the water cluster in the epoxy matrix and the epoxy-SWCNT interface. The MD simulation results indicate that in the humid environment, the water molecules are absorbed into the free volume in the cross-linked structure and the epoxy-bonded interface, which interfere with the molecular interaction inside the nanocomposite structure, resulting in the degeneration of the material performance. The observations at the nanoscale provide the structural basis for the global moisture-affected structural deterioration obtained from the macroscale experiments, such as the dynamic thermal mechanical analysis of the epoxy/organoclay and epoxy/CNT nanocomposites, as well as the lap shear tests of the epoxy/CNT composite adhesive [8,11,13]. This paper uses the nanoscale simulation to explain the global structural degradation of the epoxy/CNT nanocomposite, which leads to the understanding of the relationship between the molecular interactions and the material structure and properties with various moisture concentrations.

## 3. Materials and Methods 

In this study, the simulation approaches include the modeling of the epoxy/SWCNT nanocomposite by using Materials Studio software [57], and the structural relaxation and the dynamic deformation of the nanocomposite model in the open source code LAMMPS [58]. The interactions inside the epoxy matrix and between the epoxy and the SWCNT are described by the consistent valence force field (CVFF) [59,60], as it has been extensively used in investigating the epoxy and the epoxy/CNT nanocomposite, which yields good agreements with the theoretical and experimental results [19,20,39,61]. Meanwhile, the interactions between the carbon atoms in the SWCNT are defined with the adaptive intermolecular reactive empirical bond-order (AIREBO) potential, which is parameterized against the density functional theory calculations and experimental results [62], and its applicability in the investigation of the CNT in the polymer nanocomposite has been validated in recent studies [63,64]. The parameters of the TIP3P model are used to simulate the water molecule [65], and its bond length and angle are kept constant during the simulation by using the SHAKE algorithm [66]. The van der Waals (vdW) and the short-range Coulombic interactions between the non-bonded atoms are calculated with a cutoff distance of 10 Å, which is normally used in the investigation of the epoxy and epoxy/CNT composites [19,20,34,38,61], and the long-range Coulombic interaction is treated with the particle-particle particle-mesh (PPPM) solver [67]. Partial charges of the atoms are determined by using the bond increment method [68]. The detailed simulation method used in this work is provided in the subsequent sections.

### 3.1. Atomistic Models

In the modeling process, the initial epoxy/SWCNT composite structure is constructed by placing a SWCNT segment into the simulation cell, and by randomly distributing the epoxy monomers in the available space, as shown in Figure 1. As a single SWCNT segment is the basic unit of the agglomeration of the CNTs, it is chosen as the filler in the nanocomposite structure. Similar configuration of a single CNT embedded in the polymer matrix is considered as the representative model of the nanocomposite used in the simulation studies [35,36]. A total of thirty-eight epoxy monomers (7562 atoms) are packed in the periodic simulation cell with a density of 1.07 g·cm^−3^, which is based on the commercially-available data. After that, the initial equilibration is carried out on the uncross-linked structure. Subsequently, the cross-linking process of the epoxy matrix is performed by adopting a cross-linking algorithm, which involves the structural relaxation after each cross-linking reaction, so that the epoxy matrix can get rid of the serious geometrical distortion. The detailed steps of the cross-linking process are described in previous studies and in the Supplementary Materials (Figure S7) [19,20]. The constructed epoxy matrix possesses the maximum cross-linking density of 81%, which is in the range of the synthesized polymer in the experiment [69,70]. After the cross-linking process, the nanocomposite model is equilibrated by adopting an equilibration scheme, which consists of the pressure control to significantly improve the accuracy of the achieved density [19,20]. A final equilibration process is carried out in the isothermal and isobaric (NPT) ensemble at a constant temperature of 300 K and a constant pressure of 1 atm for 8 ns. Before the equilibration is completed, the root mean squared displacement (RMSD) of the atoms remains at a constant level, which implies that the model is equilibrated properly.

### 3.2. Equilibration and Tensile Deformation

After the model construction, the amount of 1.0 wt% water molecules is firstly added in the model, which is then equilibrated to reach the equilibrium state. Accordingly, the model is equilibrated in the NPT ensemble for 5 ns. The fully equilibrium state is confirmed by examining the RMSD of the atoms. Based on the equilibrated structure with 1.0 wt% moisture concentration, another of 1.0 wt% water molecules is added to the model for the condition with the concentration of 2.0 wt%. A 5 ns equilibration process is carried out on the nanocomposite model. Similarly, the amount of 1.0 wt% water molecules is added to the equilibrated model successively for the condition with the concentration of 3.0 and 4.0 wt%. After the structural equilibration, the models with various moisture concentrations are subjected to the computational tensile deformation to characterize the moisture-affected mechanical behavior. During the tensile deformation, one direction of the simulation cell containing the model is deformed in a step-wise manner and the atmospheric pressure in the two transverse directions is maintained. The deformation is applied at a strain rate of 1 × 10^8^ s^−1^, which is typically used for obtaining the system response within a reasonable timespan [19,20]. Each orthogonal direction is subjected to the tensile test for three times, and for all the deformation, the nanocomposite model is deformed by 3.0% in total. The reported stress-strain data are the averaged results from all the simulation runs. Furthermore, the nanocomposite models with the moisture concentrations of 0.0 and 4.0 wt% are deformed to a strain of 25.0% to quantify the moisture effect on the nanocomposite plastic deformation. The tensile deformation is carried out along the direction parallel to the SWCNT axis for three times, and the recorded data are averaged for the reported simulation results.

## 4. Conclusions

In this work, the moisture effect on the structure and properties of the epoxy/SWCNT nanocomposite is explored at the nanoscale by using MD simulations. The nanocomposite model is constructed with a SWCNT segment embedded in the highly cross-linked epoxy matrix, which possesses the structural characteristic in good accord with various experimental and simulation observables, and its structure and properties are affected significantly by the addition of water molecules. Due to the electrophilic nature, the added water molecule has a strong affinity for the epoxy hydroxyl groups, while its affinity for the hydrophobic SWCNT is low, and the difference in the distribution and the structure of the water molecules affects the molecular interactions inside the structure. At the low moisture concentration, the water molecules predominantly form the water-nanocomposite H-bonds and small clusters, i.e., the Type I water molecule and cluster acting as the important plasticizer and the Type II water molecule and cluster bridging several functional groups and, thus, the elastic properties of the nanocomposite only deteriorate slightly. As the moisture concentration increases, the added water molecules begin to form a large number of the water-water H-bonds and the large clusters located in the vicinity of the epoxy functional groups and in the available space close to the epoxy-SWCNT interface, which significantly disrupt the molecular interactions inside the structure and lead to the softening of the network. Furthermore, with the existence of the water molecules, they affect the interaction between the epoxy and the SWCNT, which deteriorates the nanocomposite performance at the plastic deformation regime.

The interplay between the water molecules and the structure and properties of the epoxy/SWCNT nanocomposite that is investigated in this work is useful for predicting the durability of the nanocomposite, as the polymer composite tends to absorb the moisture during the intended service life. The simulation results suggest that the water clusters at the high moisture concentration, especially those close to the epoxy-SWCNT interface, lead to the local structural swelling and the plasticization of the mechanical properties, which can deteriorate the global behavior of the nanocomposite material, and reduce its service life. Therefore, the environment humidity level should be under a constant control to prevent the excess moisture diffusion to the epoxy-based nanocomposite, especially for those used in the load-bearing applications, where the moisture-induced ageing can be more dangerous. This work shows the applicability of the MD simulations in understanding the mechanisms of the moisture-affected behaviors of the nanocomposite which has been commonly observed in the applications at larger length scales. It is envisioned that this work will be beneficial to the design, manufacturing, and engineering applications of the polymer composite with an enhanced moisture-resistant property.

## Supplementary Materials

The following are available online at http://www.mdpi.com/2079-4991/7/10/324/s1, Figure S1: The stress-strain data of the nanocomposite model obtained in this study, in comparison to those of the neat epoxy obtained from the experiment and simulation [41,42], Figure S2: The radial distribution function (RDF) between the oxygen of the functional groups and the carbon of the single-walled carbon nanotube (SWCNT) with respect to the moisture concentration: (**a**) the oxygen of the hydroxyl groups; and (**b**) the ether oxygen, Figure S3: The RDF between the oxygen of the functional groups and the oxygen of the water molecule for the neat epoxy with respect to the moisture concentration [42]. The RDFs for the water-hydroxyl interactions display higher peaks than those for the water-ether interactions, as demonstrated in the enlarged picture. Meanwhile, the intensity of the peak decreases monotonically with the increasing moisture concentration for both water-functional group interactions, Figure S4: After the equilibration run, the two-dimensional distribution map of the water molecules in the plane perpendicular to the SWCNT axis with respect to the moisture concentration: (**a**) 1.0 wt%; (**b**) 2.0 wt%; (**c**) 3.0 wt%; (**d**) 4.0 wt%. The red dot denotes one atom, and the black dot denotes two atoms at the particular location. For clarity, the SWCNT is labeled by the solid circle and the cutoff distance from the SWCNT surface is labeled by the dashed circle, Figure S5: The distribution of the hydrogen bond (H-bond) probability for a given water cluster in the epoxy/SWCNT nanocomposite with respect to the moisture concentration: (**a**) 1.0 wt%; (**b**) 2.0 wt%; (**c**) 3.0 wt%; (**d**) 4.0 wt%. The single water clusters do not form the H-bond, the Type I water clusters form one water-nanocomposite H-bond, and the Type II water clusters form more than one water-nanocomposite H-bond, Figure S6: The mean squared displacement (MSD) of the water molecules as a function of the simulation time in the epoxy/SWCNT nanocomposite with respect to the moisture concentration: with the increasing concentration, the diffusion of the water molecules inside the structure is restricted, Figure S7: Procedures of the cross-linking process [19]: (**a**) the oxygens in the epoxide groups are treated as the potential reactive oxygens O (labeled in red), and the carbons in the methylene bridge of the epoxide groups are treated as the potential reactive carbons C (labeled in blue); when the distance between the O and C of different epoxide groups (indicated by the dashed line) is less than the reaction radius (indicated by the dashed circle), they are recognized as the reactive pair; (**b**) the two epoxide groups comprising the recognized reactive pair are open by removing the bond between the oxygen and the carbon in the methylene bridge of each epoxide group, respectively; (**c**) the recognized reactive pair O and C of the two open epoxide groups are connected by a newly created bond to form the cross-link between the two monomers; (**d**) the unreacted atoms in the two open epoxide groups are saturated with the hydrogen.
