# Supplementary material for: Molecular Mechanics of the Moisture Effect on Epoxy/Carbon Nanotube Nanocomposites
Source: Nanomaterials (Basel). 2017 Oct 13;7(10):324. doi: 10.3390/nano7100324 (PMC5666489; doi:10.3390/nano7100324)
Supplement: Supplementary file 1 [file nanomaterials-07-00324-s001.pdf]

Supplementary Materials

Molecular Mechanics of the Moisture Effect on Epoxy/Carbon Nanotube Nanocomposites

Lik-ho Tam and Chao Wu *

School of Transportation Science and Engineering, Beihang University, 37 Xueyuan Road, Beijing 100191, China; leo_tam@buaa.edu.cn

* Correspondence: wuchao@buaa.edu.cn; Tel.: +86-010-8233-9923

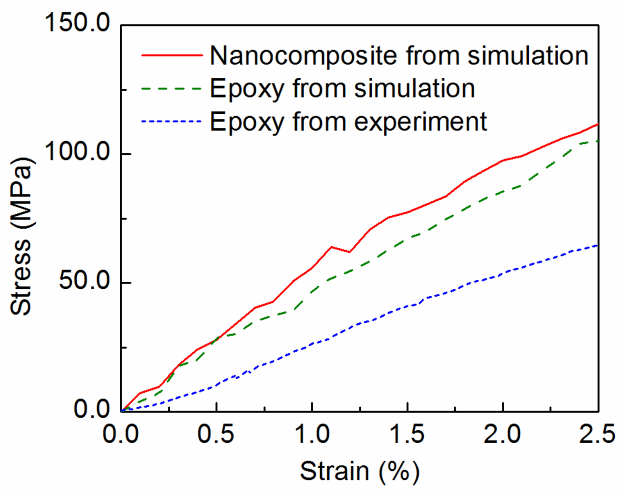

Figure S1. The stress-strain data of the nanocomposite model obtained in this study, in comparison to those of the neat epoxy obtained from the experiment and simulation [41,42].

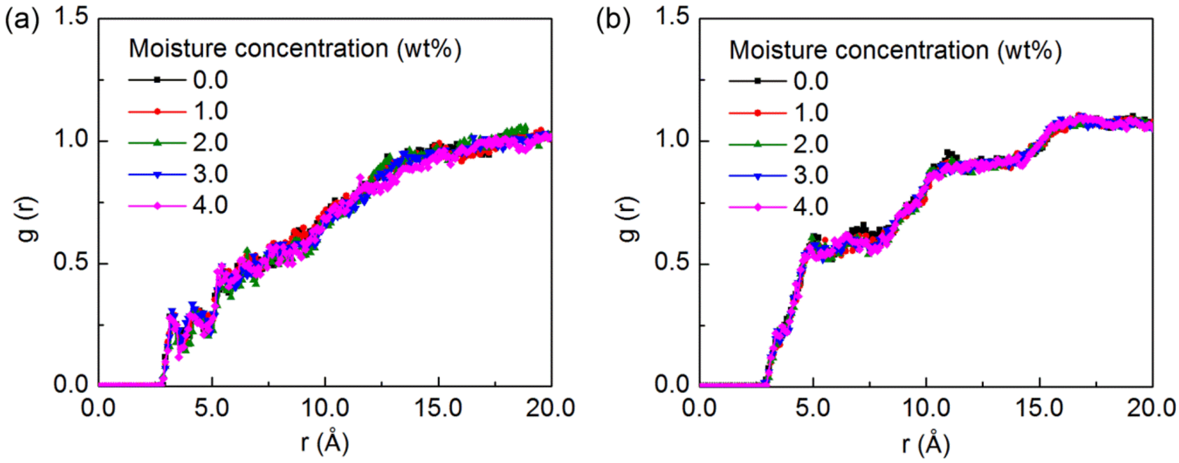

Figure S2. The radial distribution function (RDF) between the oxygen of the functional groups and the carbon of the single-walled carbon nanotube (SWCNT) with respect to the moisture concentration: (**a**) the oxygen of the hydroxyl groups; and (**b**) the ether oxygen.

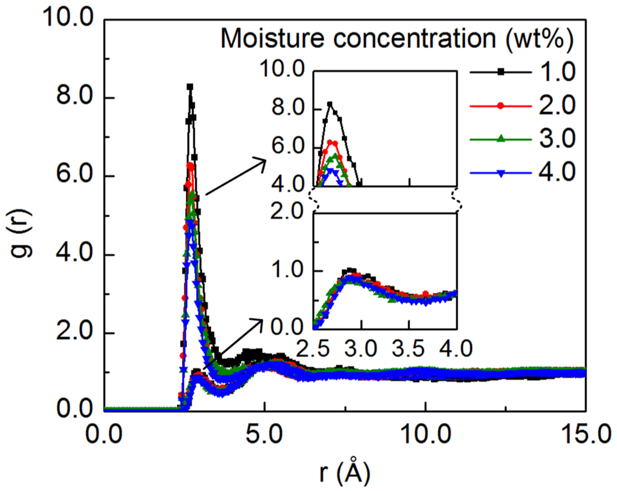

Figure S3. The RDF between the oxygen of the functional groups and the oxygen of the water molecule for the neat epoxy with respect to the moisture concentration [42]. The RDFs for the water-hydroxyl interactions display higher peaks than those for the water-ether interactions, as demonstrated in the enlarged picture. Meanwhile, the intensity of the peak decreases monotonically with the increasing moisture concentration for both water-functional group interactions.

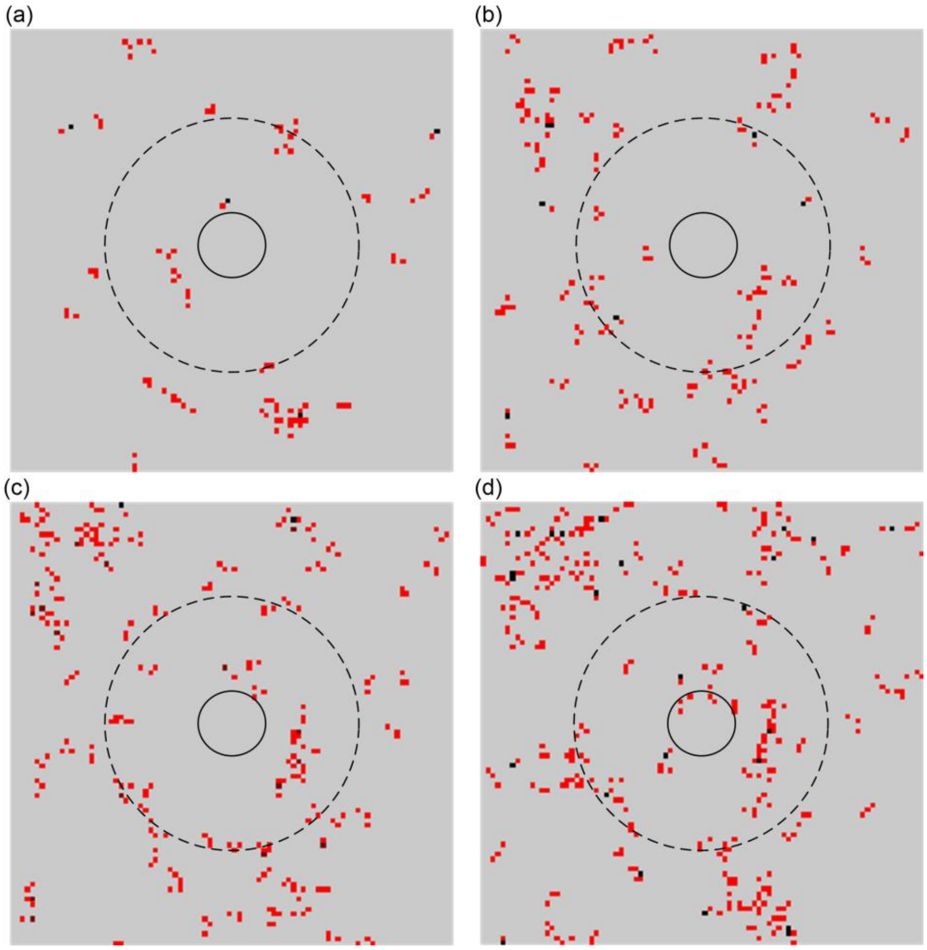

Figure S4. After the equilibration run, the two-dimensional distribution map of the water molecules in the plane perpendicular to the SWCNT axis with respect to the moisture concentration: (**a**) 1.0 wt%; (**b**) 2.0 wt%; (**c**) 3.0 wt%; (**d**) 4.0 wt%. The red dot denotes one atom, and the black dot denotes two atoms at the particular location. For clarity, the SWCNT is labeled by the solid circle and the cutoff distance from the SWCNT surface is labeled by the dashed circle.

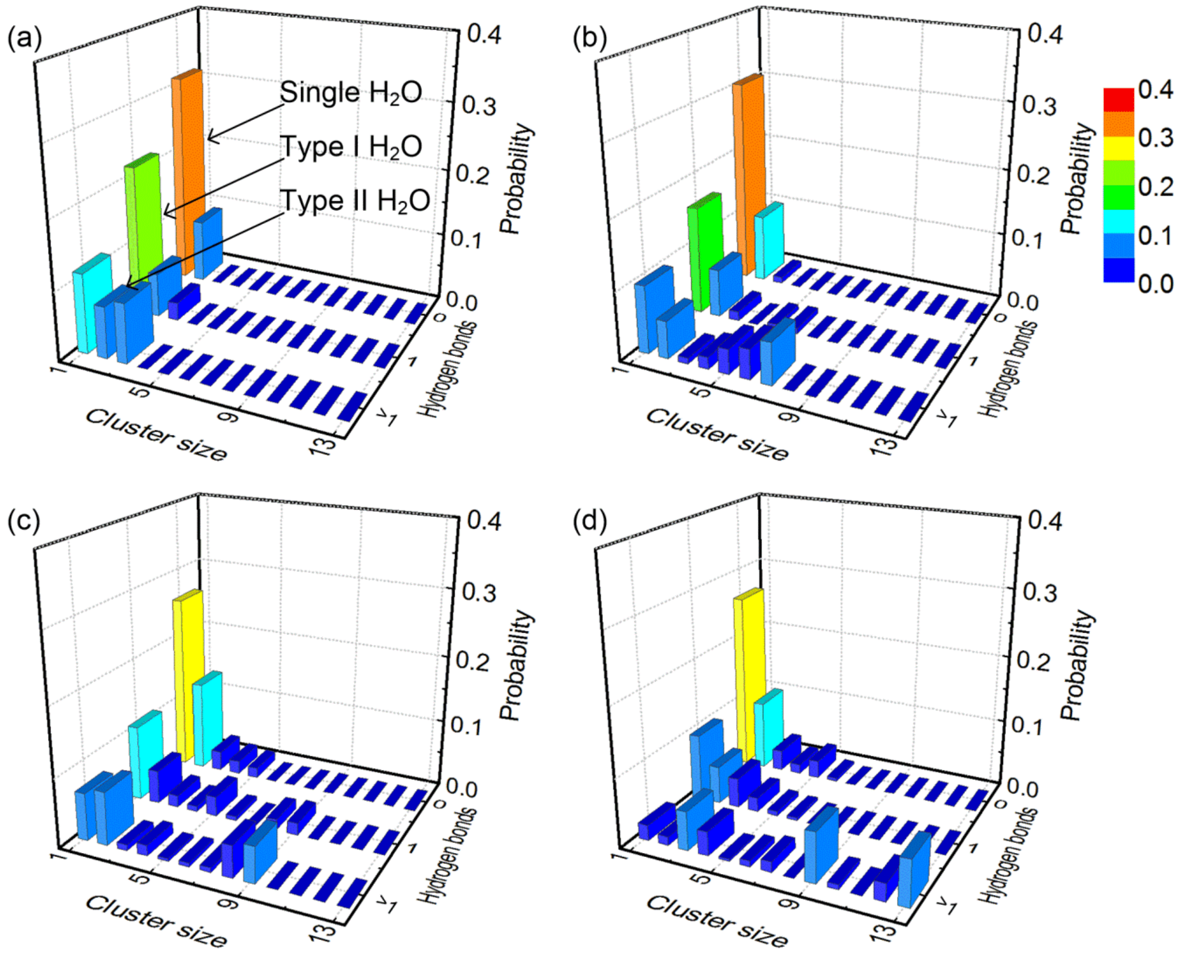

Figure S5. The distribution of the hydrogen bond (H-bond) probability for a given water cluster in the epoxy/SWCNT nanocomposite with respect to the moisture concentration: (**a**) 1.0 wt%; (**b**) 2.0 wt%; (**c**) 3.0 wt%; (**d**) 4.0 wt%. The single water clusters do not form the H-bond, the Type I water clusters form one water-nanocomposite H-bond, and the Type II water clusters form more than one water-nanocomposite H-bond.

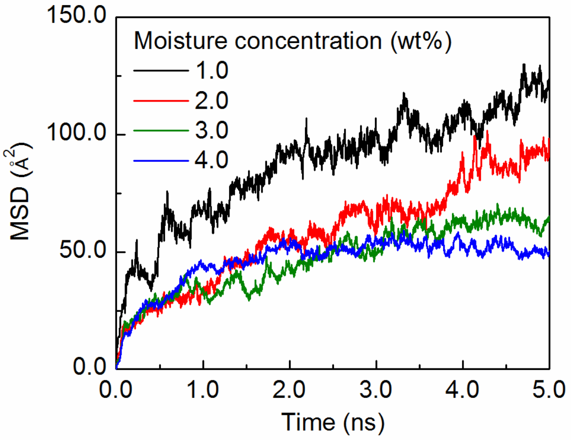

Figure S6. The mean squared displacement (MSD) of the water molecules as a function of the simulation time in the epoxy/SWCNT nanocomposite with respect to the moisture concentration: with the increasing concentration, the diffusion of the water molecules inside the structure is restricted.

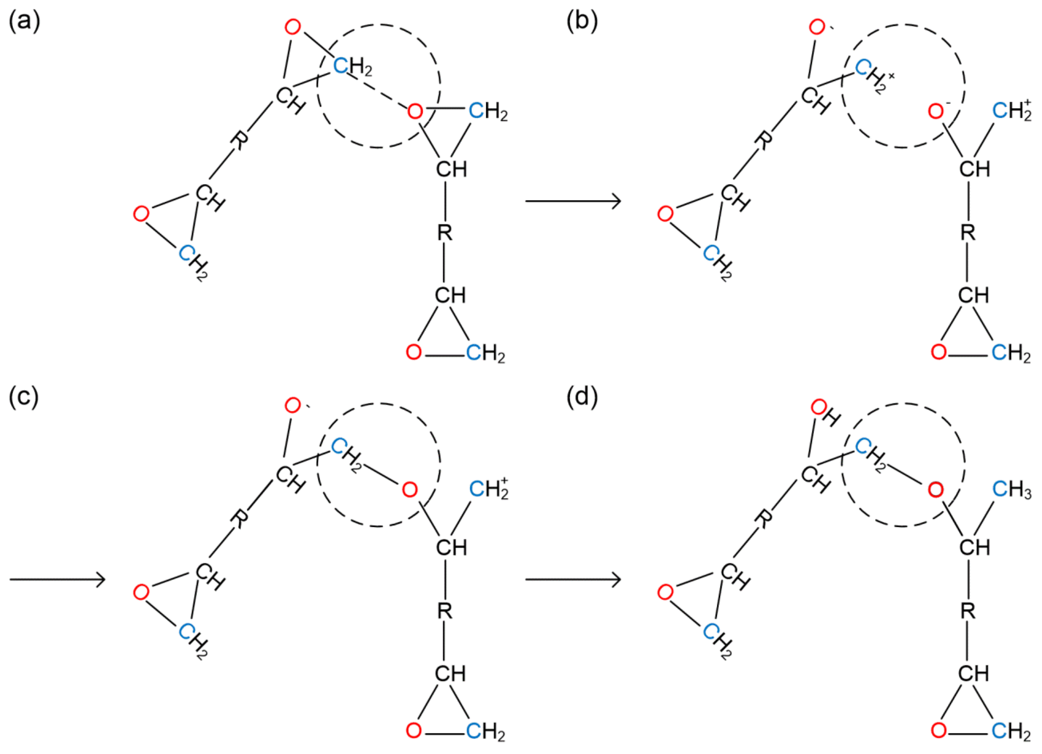

Figure S7. Procedures of the cross-linking process [19]: (**a**) the oxygens in the epoxide groups are treated as the potential reactive oxygens O (labeled in red), and the carbons in the methylene bridge of the epoxide groups are treated as the potential reactive carbons C (labeled in blue); when the distance between the O and C of different epoxide groups (indicated by the dashed line) is less than the reaction radius (indicated by the dashed circle), they are recognized as the reactive pair; (**b**) the two epoxide groups comprising the recognized reactive pair are open by removing the bond between the oxygen and the carbon in the methylene bridge of each epoxide group, respectively; (**c**) the recognized reactive pair O and C of the two open epoxide groups are connected by a newly created bond to form the cross-link between the two monomers; (**d**) the unreacted atoms in the two open epoxide groups are saturated with the hydrogen.

References

19. Tam, L.-h.; Lau, D. A molecular dynamics investigation on the cross-linking and physical properties of epoxy-based materials. *RSC Adv.* **2014**, *4*, 33074–33081.
41. Feng, R.; Farris, R.J. The characterization of thermal and elastic constants for an epoxy photoresist SU8 coating. *J. Mater. Sci.* **2002**, *37*, 4793–4799.
42. Tam, L.-h.; Lau, D.; Wu, C. Understanding the moisture effect on the cross-linked epoxy via molecular dynamics simulations. *J. Mol. Model.* (under review).
